# The wrinkles of the soul- a psychoneuroimmunological approach to aging

**DOI:** 10.1192/j.eurpsy.2023.2109

**Published:** 2023-07-19

**Authors:** B. A. Oroian, G. I. Costandache, A. Salaru, P. F. Ionescu, P. Nechita

**Affiliations:** Institute of Psychiatry “Socola” Iași, Iasi (0 km), Romania

## Abstract

**Introduction:**

Psychoneuroimmunology is a discipline that has emerged over the past decades as a broad interdisciplinary field that closely observes the relationship between the psychological state, the nervous system, the endocrine system, and the immune system. The natural aging process leads to alterations in the immune functions, involving lymphocyte dysregulation, and therefore leading to a higher risk of developing coronary artery diseases, infectious diseases or even cancer.

**Objectives:**

The aim of our inquiry is to evaluate the existing body of work with a focus on studies that observed the intricate connections between psychosocial factors and immunity.

**Methods:**

We performed a systematic review on PubMed and a targeted literature search concentrating on all the factors involved in immunosenescence and their consequences.

**Results:**

The causality between emotional stressors (acute or chronic), lack of social support, adverse life events, coping mechanisms, personality traits, as well as endocrine changes and multiple age-related pathologies is often undeniable. Other relevant factors include nutrition, sleep, physical activity and substance use. As people grow older, they face a number of psychosocial stressors, such as retirement, social isolation, loss of independence, low income, a decrease in productivity and also somatic comorbidities. These factors, together with an age-related decline in immune function, can constitute not only a psychosocial disadvantage of the elderly, but also a risk factor able to trigger further deterioration of the immune system.

**Conclusions:**

Age-associated alterations of the immune response represent a complex concept. Given that we are dealing with the phenomenon of aging in the general population, the field of psychoneuroimmunology can make a significant contribution in establishing the different mechanisms through which seniors can cushion the impact of stressors in regards to health and illness. Therefore, we can pave the way for an individualized approach and support for patients, as well as provide better therapeutic outcomes.

**Disclosure of Interest:**

None Declared

